# Lumen-apposing metal stent in a right renal cyst as a complication of endoscopic ultrasound-guided gallbladder drainage

**DOI:** 10.1055/a-2744-8771

**Published:** 2025-12-08

**Authors:** Satoshi Ito, Toru Okuzono, Hiroaki Saito, Jyunichi Togashi, Tomoki Matsuda

**Affiliations:** 1 26387 Department of Gastroenterology, Sendai Kosei Hospital, Sendai, Japan

Although endoscopic ultrasound-guided gallbladder drainage (EUS-GBD) is considered a relatively safe technique, adverse events have been reported 1 2 . We present a case of lumen-apposing metal stent (LAMS) maldeployment in a right renal cyst as a complication of EUS-GBD.

A 72-year-old woman underwent endoscopic retrograde cholangiopancreatography with the placement of a plastic stent in the left intrahepatic bile duct for hilar cholangiocarcinoma. Four days later, she developed epigastric pain. Computed tomography (CT) revealed gallbladder enlargement and increased pericholecystic fat attenuation, leading to acute cholecystitis ( Fig. 1 **a** ). Given the unresectable cholangiocarcinoma, cholecystectomy was not indicated and EUS-GBD using LAMS was performed ( Video 1 ).

**Fig. 1:**
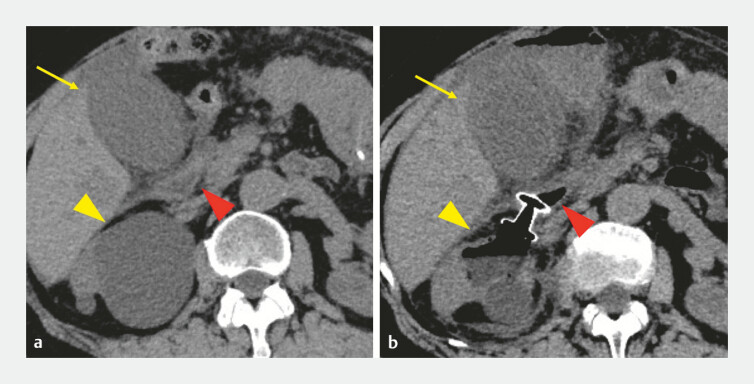
**a** A computed tomography scan revealing acute cholecystitis (yellow arrow), a renal cyst (yellow arrowhead) near the gallbladder, and the duodenal bulb (red arrowhead). **b** A computed tomography scan showing the exacerbation of cholecystitis (yellow arrow) and the maldeployment of a LAMS in a right renal cyst (yellow arrowhead).

Maldeployment of a lumen-apposing metal stent in a right renal cyst as a complication of endoscopic ultrasound-guided gallbladder drainage.Video 1

A linear echoendoscope (GF-UCT 260; Olympus Medical Systems, Tokyo, Japan) was advanced into the duodenal bulb, and revealed a 60 mm × 60 mm fluid-filled lesion. A LAMS (Hot AXIOS; Boston Scientific Corporation, Marlborough, MA, USA) was successfully deployed into the area. However, the patient later developed worsening abdominal pain and elevated levels of inflammatory markers. A repeated CT scan revealed that the LAMS had been deployed between the duodenal bulb and the right renal cyst, and the cholecystitis had not resolved ( Fig. 1 **b** ). Emergency percutaneous transhepatic gallbladder drainage was performed, and her abdominal symptoms and inflammatory markers improved, allowing the resumption of oral intake on day 4, and discharge on day 10. One month later, the LAMS placed in the right renal cyst was successfully removed without adverse events.

The risk of misidentifying a hepatic or renal cyst as a gallbladder during EUS-GBD has been previously reported 3 4 . In our case, a thick-walled gallbladder was visualized in the stomach ( Fig. 2 **a** ), whereas the renal cyst visualized in the duodenum showed no wall thickening or internal debris ( Fig. 2 **b** ). Careful EUS assessments should be performed, considering the possibility that the right renal cyst may be punctured from the duodenal bulb.

**Fig. 2:**
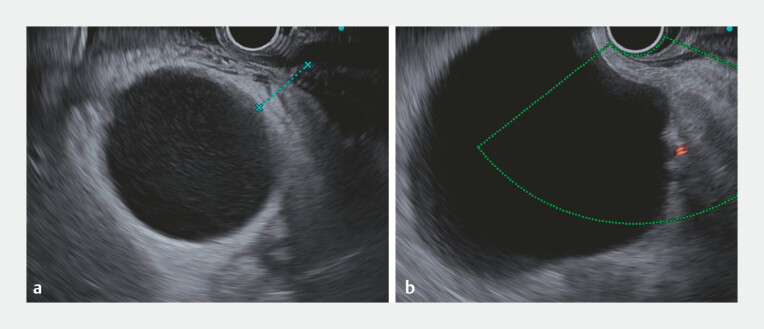
**a** An endoscopic ultrasound image from the stomach showing a thick-walled gallbladder with internal debris. **b** An endoscopic ultrasound image from the duodenal bulb showing a renal cyst without wall thickening or internal debris.

Endoscopy_UCTN_Code_TTT_1AS_2AJ

## Notes

Endoscopy E-Videos https://eref.thieme.de/e-videos
*E-Videos* is an open access online section of the journal *Endoscopy* , reporting on interesting cases and new techniques in gastroenterological endoscopy. All papers include a high-quality video and are published with a Creative Commons CC-BY license. Endoscopy E-Videos qualify for HINARI discounts and waivers and eligibility is automatically checked during the submission process. We grant 100% waivers to articles whose corresponding authors are based in Group A countries and 50% waivers to those who are based in Group B countries as classified by Research4Life (see: https://www.research4life.org/access/eligibility/ ). This section has its own submission website at https://mc.manuscriptcentral.com/e-videos .
